# Chlorantraniliprole against the black cutworm *Agrotis ipsilon* (Lepidoptera: Noctuidae): From biochemical/physiological to demographic responses

**DOI:** 10.1038/s41598-019-46915-0

**Published:** 2019-07-17

**Authors:** Falin He, Shiang Sun, Haili Tan, Xiao Sun, Chao Qin, Shoumin Ji, Xiangdong Li, Jiwang Zhang, Xingyin Jiang

**Affiliations:** 1 Key Laboratory of Pesticide Toxicology and Application Technique, College of Plant Protection, Shandong Agricultural University, Tai’an, Shandong 271018 China; 2 Research Center of Pesticide Environmental Toxicology, Shandong Agricultural University, Tai’an, Shandong 271018 China; 3 Shandong Provincial Key Laboratory of Agricultural Microbiology, College of Plant Protection, Shandong Agricultural University, Tai’an, Shandong 271018 China; 4 State Key Laboratory of Crop Biology, College of Agronomy, Shandong Agricultural University, Tai’an, Shandong 271018 China

**Keywords:** Non-model organisms, Non-model organisms, Biocatalysis, Biocatalysis

## Abstract

*Agrotis ipsilon* (Lepidoptera: Noctuidae) is a major underground pest that damages many agricultural crops in China and other countries. A diet-incorporation-based bioassay was conducted to evaluate the sublethal effects of the novel anthranilic diamide chlorantraniliprole on the nutritional physiology, enzymatic properties and population parameters of this cutworm. Chlorantraniliprole exhibited signs of active toxicity against third instar larvae of *A*. *ipsilon*, and the LC_50_ was 0.187 μg.g^−1^ of artificial diet after treatment for 72 h. The development time of the larval, pupal and adult stages was significantly affected after chlorantraniliprole exposure, compared to the control treatment. Relative to the control treatment, chlorantraniliprole decreased pupal and adult emergence rates, fecundity and fertility and increased the proportions of developmental deformities, the adult preoviposition period (APOP) and the total preoviposition period (TPOP). Furthermore, compared to those treated with the control, *A*. *ipsilon* larvae treated with low doses of chlorantraniliprole decreased food utilization and nutrient content (protein, lipid, carbohydrate, trehalose), showed lower pupal weights and growth rates. Compared with the control treatment, chlorantraniliprole significantly reduced digestive enzyme activities and observably increased detoxifying and protective enzyme activities and hormone titers. Importantly, these chlorantraniliprole-induced changes affected life table parameters of the cutworm. These results suggest that chlorantraniliprole at low concentrations can impair *A*. *ipsilon* development duration, normal food consumption and digestion process, enzymatic properties, hormone levels, fecundity and population levels. Chlorantraniliprole exhibit the potential to be exploited as a control strategy for this cutworm.

## Introduction

The black cutworm (BCW), *Agrotis ipsilon* (Lepidoptera: Noctuidae), is widely distributed in many countries and regions worldwide^1,2^. *A. ipsilon* is one of the most dangerous species of underground pests and can feed on more than 100 types of host plants (e.g., corn, wheat, cotton, soybean, vegetables and a variety of weeds^3,4^. One larva may bite off several plant seedlings in a night^5^. In particular, *A. ipsilon* larvae can cause serious damage at the fourth-sixth and/or higher instar stages^5^. BCW can cause hidden damage in fields, and it is difficult to fully expose the pests to insecticides by spraying, which results in reduced effectiveness of control strategies^6^. Currently, the selection of high-efficiency insecticides and suitable application methods is an important problem in the development of integrated pest management (IPM) strategies for controlling the black cutworm.

Chlorantraniliprole is a novel anthranilic diamide insecticide developed by DuPont Crop Protection that has been registered and effectively used for the control of many lepidopteran pests and non-lepidopteran species, such as Coleoptera, Diptera, Hemiptera and Isoptera species in various crops^7,8^. Chlorantraniliprole has a unique mode of action, activating the unregulated release of internal calcium stores, which leads to Ca^2+^ depletion, feeding cessation, lethargy, muscle paralysis, and insect death^9^. This insecticide is a useful alternative to the more toxic conventional insecticides because it is environmentally friendly and exhibits relatively low toxicity against nontarget animals in Integrated Pest Management (IPM) programs^10,11^.

In addition to having a direct killing effect on target pests exposed to lethal doses of insecticide^12,13^, also results, to a certain extent, in effects on insect physiology, biology, behavior, reproduction, longevity and so on, of survival individuals exposed to low-lethal doses of insecticide, due to the continuous degradation and variable distributions of chemicals when insecticides application in fields^14,15^. That is, chemicals may generate sublethal effects on target or nontarget insects when they are exposed to insecticides at low-lethal concentrations^16,17^, which can stimulate the rate of development and adult fecundity of targeted insects^18,19^, Individual differences in toxicity and sublethal effects are related to exposure time and dose of insecticide^20^. In addition, sublethal effects vary among different classes of insecticides and different species of targeted insects^21^.

The age-stage, two-sex life table, which can fully describe population dynamics in a comprehensive manner and can also explain the multiple sublethal effects on targeted insects^22,23^. However, these studies have only paid attention to population parameters, and other physiological and/or biochemical characteristics should also be considered after exposure to low concentrations of insecticides. Changes in bioactivity can be used to assess and predict the toxicity and potential efficacy of insecticides in the control of insect pests^24,25^. Hence, studying the sublethal effects of insecticides on targeted insect pests is crucial to guide the scientific application of insecticides.

The objective of this study was to obtain a comprehensive understanding of the sublethal effects of chlorantraniliprole on *A. ipsilon*. Based on the toxicity of chlorantraniliprole against third instar larvae, we investigated its sublethal effects on the development, population parameters and bioactivity parameters (nutritional indices; nutrient content; digestive, detoxifying and protective enzyme activities; and hormone titers) of *A. ipsilon* when third instar larvae were exposed to chlorantraniliprole at the LC_05_, LC_25_ and LC_45_ concentrations. The results may help us understand the sublethal effects of chlorantraniliprole on the population dynamics and bioactivity of *A. ipsilon* and provide important information about the rational application of chlorantraniliprole in IPM strategies for *A. ipsilon*.

## Results

### Toxicity of chlorantraniliprole against third instar larvae of *A. ipsilon*

This experiment determined the toxicity of chlorantraniliprole against third instar larvae of *A. ipsilon* (Table 1). The results showed that chlorantraniliprole exhibited active toxicity against *A. ipsilon* larvae. After 72 h of treatment, for the artificial diet, the LC_50_ was 0.187 μg.g^−1^, and the LC_05_, LC_25_, and LC_45_ were 0.007 μg.g^−1^, 0.048 μg.g^−1^, and 0.145 μg.g^−1^, respectively.

**Table 1:** Toxicity of chlorantraniliprole to 3rd-instar larvae of Agrotis ipsilon.

Insecticide	N^a^	Slope ± SE	LC_50_ (95%CL)^b^ (μg.g^−1^)	LC_45_ (95%CL)^b^ (μg.g^−1^)	LC_25_ (95%CL)^b^ (μg.g^−1^)	LC_05_ (95%CL)^b^ (μg.g^−1^)	*χ*^2^ (*df*)^c^	*p-*value^c^
chlorantraniliprole	840	2.582 ± 0.597	0.187 (0.153–0.229)	0.145 (0.116–0.182)	0.048 (0.034–0.069)	0.007(0.004–0.013)	1.385 (6)	0.987

^a^Number of larvae.

^b^95% confidence limits.

^c^Chi-square value (*χ*^2^), degrees of freedom (*df*), and *p*-value as calculated by probit analysis with SPSS 23.0.

### Effects of chlorantraniliprole on the growth and development of *A. ipsilon*

The sublethal effects of chlorantraniliprole on the growth and development parameters of *A. ipsilon* were determined (Table 2). Chlorantraniliprole at low-lethal concentrations did significantly shorten the development time of third and fourth instar lavae of *A. ipsilon*. Larval duration of fifth/sixth-instar was significantly prolonged compared to the control when third instar larvae of *A. ipsilon* were exposed to chlorantraniliprole. After treatment with chlorantraniliprole, the development time of pupae and adult longevity were lengthened after exposure to the LC_05_ concentration; however, there was no significant difference between the treatment and the control. Chlorantraniliprole significantly increased the number of deformed pupae and adults of the parental generation and offspring eggs and the percentage of seventh-instar larvae compared to the control. However, the pupation rate and adult emergence were significantly decreased when chlorantraniliprole was applied to the third instar larvae of *A. ipsilon*. No significant effect was observed on pupa weight and sex ratio when *A. ipsilon* larvae were treated with chlorantraniliprole compared to the control. The adult preoviposition period (APOP) and total preoviposition period (TPOP) of female *A. ipsilon* insects exposed to the low-lethal chlorantraniliprole treatments were significantly affected. In addition, the LC_05_ treatment did, to some extent, lengthen the oviposition period compared to that of the control group, while the LC_25_ and LC_45_ treatments shortened the oviposition period. The LC_05_, LC_25_ and LC_45_ treatments significantly reduced fecundity (1054.555, 838.588 and 619.843 eggs/female at LC_05_, LC_25_ and LC_45_, respectively) compared with the control treatment (1210.423 eggs/female) (Table 2).

**Table 2:** Effects of chlorantraniliprole treatment on growth and development (mean ± SE) of *A. ipsilon* 3rd-instar larvae.

Biological characteristics	Control		LC_05_		LC_25_		LC_45_		*P*
	n	Mean ± SE	n	Mean ± SE	n	Mean ± SE	n	Mean ± SE	
Third-instar period (days)	150	4.121 ± 0.108 a	189	3.983 ± 0.067 ab	197	3.773 ± 0.061 b	216	3.381 ± 0.069 c	<0.0001
Fourth-instar period (days)	149	4.512 ± 0.080 a	181	4.308 ± 0.087 a	189	4.029 ± 0.088 b	205	3.817 ± 0.095 b	<0.0001
Fifth-instar period (days)	146	5.867 ± 0.078 c	172	6.384 ± 0.073 b	176	6.531 ± 0.049 ab	197	6.918 ± 0.119 a	<0.0001
Sixth-instar period (days)	143	7.498 ± 0.084 b	167	7.920 ± 0.107 a	168	7.822 ± 0.056 ab	175	8.026 ± 0.052 a	0.0020
Seven-instar larvae (%)	23	15.920 ± 0.272 c	39	23.468 ± 0.379 b	63	37.735 ± 0.276 a	61	34.148 ± 0.139 a	<0.0001
Pupation rate (%)	139	97.455 ± 0.954 a	158	94.443 ± 0.748 b	148	88.918 ± 0.674 c	142	81.075 ± 0.531 d	<0.0001
Pupae with deformities (%)	139	2.718 ± 0.035 d	158	3.349 ± 0.046 c	148	4.642 ± 0.012 b	142	5.562 ± 0.048 a	<0.0001
Female pupae period (days)	70	14.608 ± 0.161 ab	78	15.228 ± 0.237 a	73	13.823 ± 0.235 b	70	12.555 ± 0.227 c	0.0003
Male pupae period (days)	69	13.575 ± 0.132 a	80	14.085 ± 0.355 a	75	13.055 ± 0.102 ab	72	12.043 ± 0.162 b	0.0028
Female pupae weight (g)	70	0.339 ± 0.013 a	78	0.327 ± 0.009 ab	73	0.293 ± 0.019 ab	70	0.275 ± 0.016 b	0.0404
Male pupae weight (g)	69	0.392 ± 0.006 a	80	0.354 ± 0.017 a	75	0.344 ± 0.036 a	72	0.311 ± 0.007 a	0.0903
Adult emergence (%)	134	96.575 ± 1.032 a	150	94.493 ± 0.847 ab	135	91.308 ± 0.878 b	123	86.253 ± 0.990 c	<0.0001
Sex ratio (♀/(♀ + ♂)) (%)	134	50.650 ± 0.404 a	150	50.333 ± 0.599 a	135	49.180 ± 0.373 a	123	48.643 ± 0.468 a	0.0470
Adults with deformities (%)	134	3.443 ± 0.015 c	150	4.250 ± 0.017 bc	135	5.465 ± 0.026 ab	123	6.245 ± 0.017 a	<0.0001
Female adult longevity (days)	68	14.128 ± 0.182 a	75	14.458 ± 0.258 a	66	13.490 ± 0.159 a	60	12.725 ± 0.234 a	0.0667
Male adult longevity (days)	66	13.265 ± 0.263 a	75	13.325 ± 0.174 a	69	12.518 ± 0.219 a	63	11.378 ± 0.151 a	0.0746
APOP^a^ (days)	68	3.073 ± 0.079 b	75	3.285 ± 0.038 ab	66	3.815 ± 0.027 ab	60	4.378 ± 0.046 a	0.0204
TPOP^b^ (days)	68	53.750 ± 0.783 c	75	55.060 ± 0.482 bc	66	56.145 ± 0.536 ab	60	57.435 ± 0.689 a	0.0007
Oviposition period (days)	68	8.788 ± 0.096 ab	75	9.158 ± 0.202 a	66	8.673 ± 0.097 ab	60	8.148 ± 0.119 b	0.0708
Fecundity (eggs/♀)	68	1210.423 ± 28.090 a	75	1054.555 ± 46.164 b	66	838.588 ± 17.816 c	60	619.843 ± 12.393 d	<0.0001
Eggs with deformities (%)	68	5.570 ± 0.087 c	75	7.155 ±± 0.105 bc	66	10.083 ± 0.107 b	60	14.673 ± 0.203 a	<0.0001

^a^Adult preoviposition period.

^b^Total preoviposition period.

Means marked with different lowercase letters in the same row are significantly different (*P* < 0.05).

### Effects of chlorantraniliprole on population parameters of *A. ipsilon*

The effects of low-lethal chlorantraniliprole concentrations on the population parameters of *A. ipsilon* were calculated using age-stage, two-sex life tables (Table 3). The intrinsic rate of increase (*r*) and the finite rate of increase (*λ*) decreased significantly in insects exposed to three low-lethal chlorantraniliprole treatments. The net reproductive rate (*R*_0_) was significantly reduced by the chlorantraniliprole treatments. At LC_25_ and LC_45_, chlorantraniliprole significantly prolonged the mean generation time (*T*), while the LC_05_ treatment resulted in no significant difference compared with the control. The gross reproductive rate (*GRR*) under the LC_05_ and LC_45_ treatments (682.39 and 347.89 offspring/individual at LC_05_ and LC_45_, respectively) was lower than the *GRR* of the control treatment (863.24 offspring/individual); however, the LC_25_ treatment did significantly increase the *GRR* (968.64 offspring/individual) compared with the control (Table 3).

**Table 3:** Estimates of life table parameters of *A. ipsilon* when 3rd-instar larvae were exposed to low-lethal concentrations of chlorantraniliprole.

Treatments	Population parameters				
	*r* (d^−1^)	*λ* (d^−1^)	*R*_0_ (offspring/individual)	*T* (d)	*GRR* (offspring/individual)
Control	0.1211 ± 0.0016 a	1.1287 ± 0.0029 a	544.66 ± 35.6515 a	52.05 ± 0.2641 b	863.24 ± 28.4125 b
LC_05_	0.1110 ± 0.0027 b	1.1174 ± 0.0037 b	395.42 ± 28.4212 b	53.86 ± 0.1987 b	682.39 ± 35.6974 c
LC_25_	0.0981 ± 0.0019 c	1.1031 ± 0.0034 c	223.47 ± 31.0527 c	55.14 ± 0.3656 a	968.64 ± 41.7569 a
LC_45_	0.0859 ± 0.0021 d	1.0897 ± 0.0023 d	124.68 ± 22.3698 d	56.12 ± 0.2543 a	347.89 ± 32.4106 d

SEs were estimated using 200,000 bootstraps and compared with paired bootstrap tests based on the 5% significance level.

Means followed by different lowercase letters in the same column are significantly different (*P* < 0.05).

*r* = the int*r*insic rate of increase; *λ* = the finite rate of increase; *R*_0_ = the net reproduction rate; *T* = the mean generation time; *GRR* = the gross reproduction rate.

**Figure 1:**
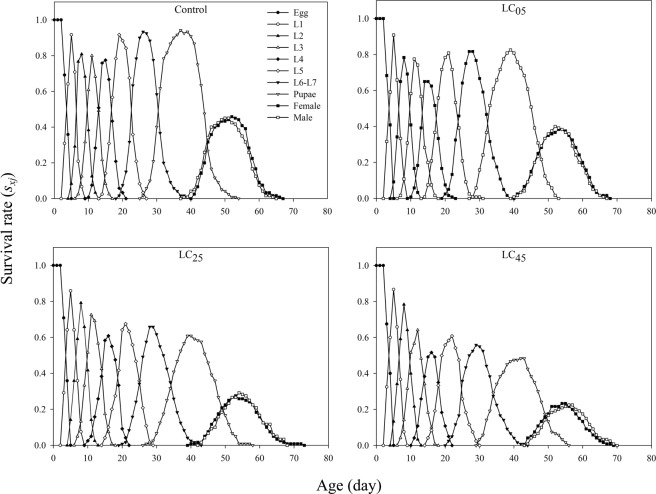
Age-stage specific survival rate (*s*_*xj*_) of *A. ipsilon* after exposure to low-lethal concentrations of chlorantraniliprole.

**Figure 2:**
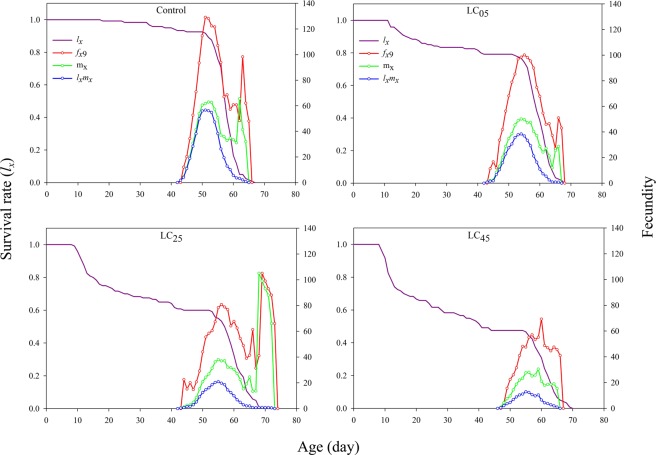
Age-specific survival rate (*l*_*x*_), female age-specific fecundity (*f*_*x*9_), age-specific fecundity of the total population (*m*_*x*_), and age-specific maternity (*l*_*x*_*m*_*x*_) of *A. ipsilon* after exposure to low-lethal concentrations of chlorantraniliprole.

The age-stage-specific survival rate (*s*_*xj*_) curve is shown in Fig. 1. Because the development rate varied among individuals, a distinct overlap phenomenon was observed between the chlorantraniliprole-treated groups and the control (Fig. 1). With increasing concentration, the probability of survival from larvae to adults significantly decreased after exposure to the LC_05_, LC_25_ and LC_45_ treatments of chlorantraniliprole (Fig. 1). The age-specific survival rate (*l*_*x*_) is the probability of newborn eggs surviving to age *x*, regardless of stage differentiation (Fig. 2). The *l*_*x*_ significantly decreased when the third instar larvae were exposed to the insecticide. The peak female age-specific fecundity (*f*_*x*9_) values under the LC_05_, LC_25_ and LC_45_ treatments were lower than that of the control treatment (Fig. 2). The age-specific fecundity of the total population (*m*_*x*_) under the LC_25_ treatment was higher than that of the control, while under the LC_05_ and LC_45_ treatments, the fecundity was lower than that of the control after exposure to chlorantraniliprole (Fig. 2). The *l*_*x*_*m*_*x*_ value is mainly dependent on *l*_*x*_ and *m*_*x*_, and the maximum *l*_*x*_*m*_*x*_ values were 51, 54, 55 and 56 days for the control, LC_05_, LC_25_ and LC_45_ treatments, respectively, while the maximum values were 56.58, 38.38, 20.87, and 12.86 for the control, LC_05_, LC_25_ and LC_45_ treatments, respectively (Fig. 2).

**Figure 3:**
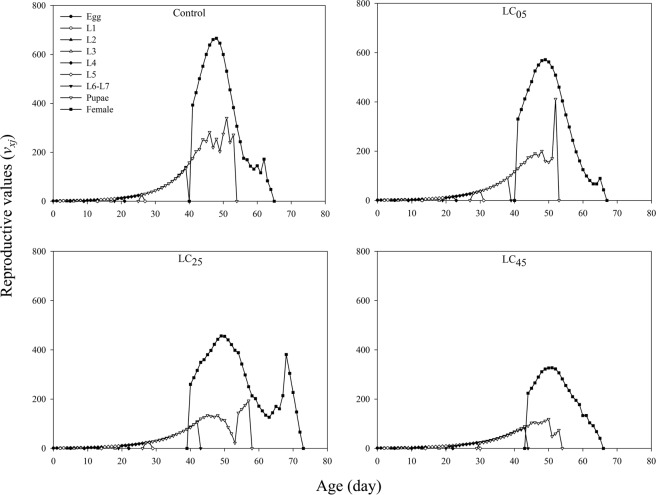
Age-stage specific reproductive value (*v*_*xj*_) of *A. ipsilon* after exposure to low-lethal concentrations of chlorantraniliprole.

The age-stage-specific reproductive value (*v*_*xj*_) curves are shown in Fig. 3. However, the *v*_*xj*_ curves for males were not produced, mainly because the contribution of males to offspring cannot be defined. There were no significant effects on the reproductive values of eggs and larvae when *A. ipsilon* larvae were treated with the insecticide. The peak reproductive values of the pupae under LC_05_ treatment were higher than that of the control, while under the LC_25_ and LC_45_ treatments, the peak was lower than that of the control. At low-lethal concentrations, chlorantraniliprole significantly decreased the female reproductive values, with peak *v*_*xj*_ values of 666.09, 571.68, 456.63, and 327.36 for the control, LC_05_, LC_25_ and LC_45_, respectively (Fig. 3).

**Figure 4:**
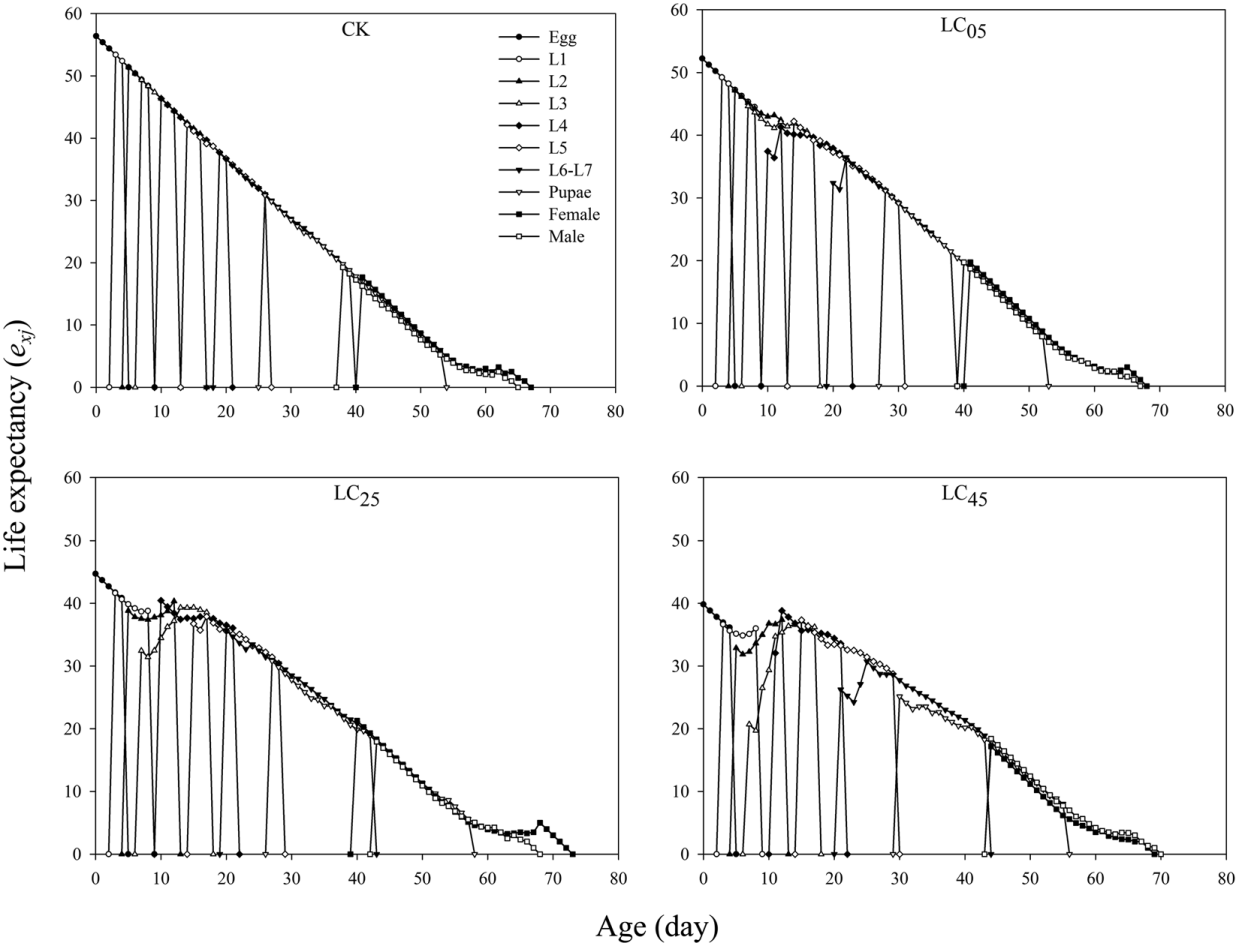
Life expectancy (*e*_*xj*_) of *A. ipsilon* after exposure to low-lethal concentrations of chlorantraniliprole.

The life expectancy (*e*_*xj*_) curves for each age-stage group of *A. ipsilon* are shown in Fig. 4. The *e*_*xj*_ value decreased gradually with age *x* because this study was carried out in the laboratory and was not adversely affected by other field conditions. At LC_05_, LC_25_, and LC_45_, chlorantraniliprole significantly decreased the *e*_*xj*_ of the egg and larval stages compared to the control group. The pupal *e*_*xj*_ dramatically decreased under the LC_45_ treatment compared to the LC_05_ and LC_25_ treatments and the control, while the *e*_*xj*_ of females decreased under the LC_25_ treatment compared to the LC_05_ and LC_45_ treatments (Fig. 4).

### Effects of chlorantraniliprole on nutritional indices

**Figure 5:**
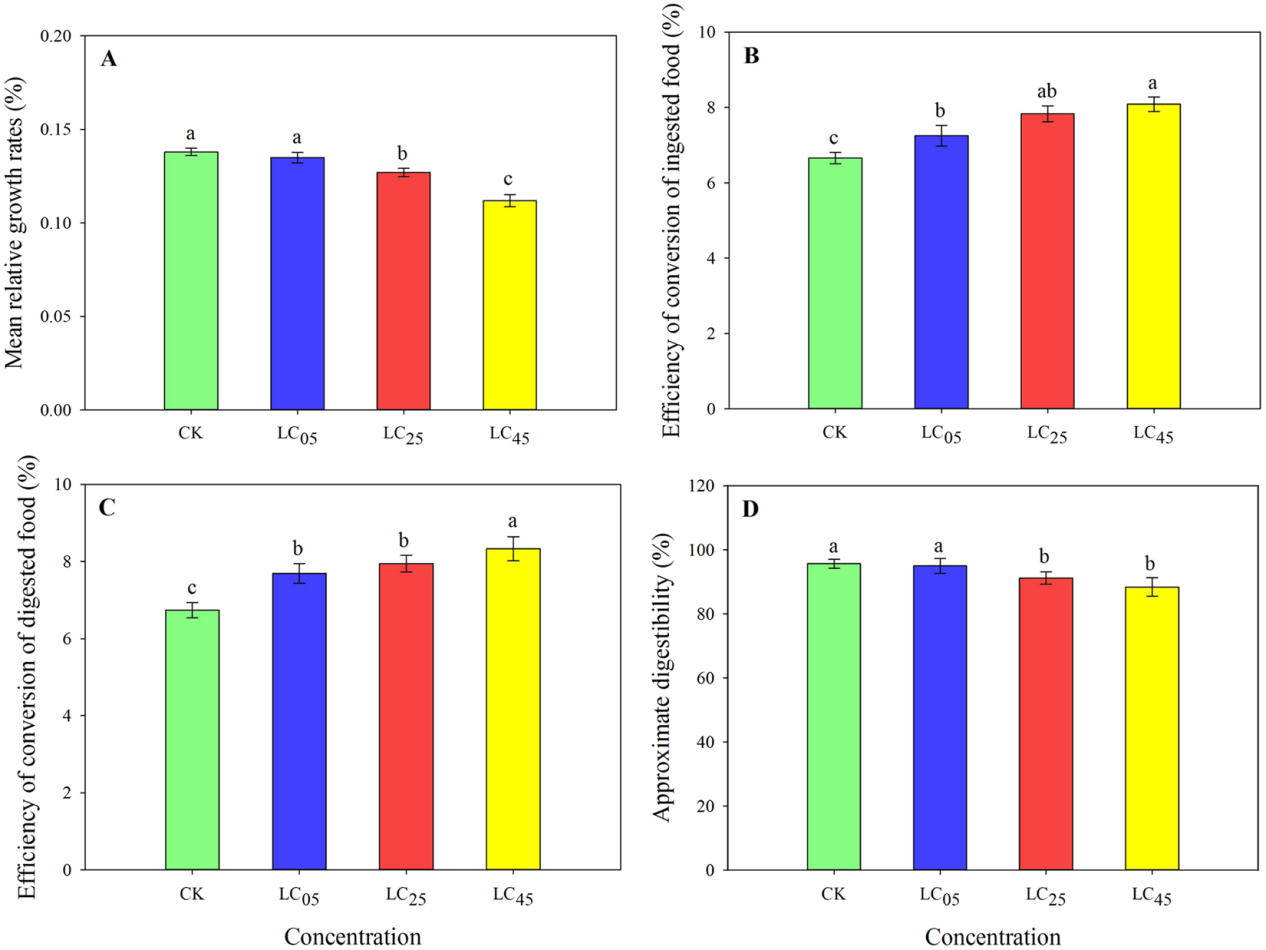
Nutritional indices (**A**) mean relative growth rates, (**B**) efficiency of conversion of ingested food, (**C**) efficiency of conversion of digested food, (**D**) approximate digestibility) of third-instar larvae of *A. ipsilon* (Mean ± SE) after exposure to low-lethal concentrations of chlorantraniliprole. Bar with the same lowercase letters show no significant differences (Student-Newman-Keuls test, *P* < 0.05).

At low-lethal concentrations, chlorantraniliprole, to a certain extent, did affect the nutritional indices of *A. ipsilon* larvae (Fig. 5). The mean relative growth rates (MRGRs) and the approximate digestibility (AD) of the third instar larvae were significantly lower after the LC_25_ and LC_45_ treatments than after the LC_05_ treatment and the control treatment (Fig. 5A,D). Nevertheless, the efficiency of conversion of ingested food (ECI) and the efficiency of conversion of digested food (ECD) were much higher after exposure to the three low-lethal concentrations of chlorantraniliprole than after exposure to the control (Fig. 5B,C).

### Effects of chlorantraniliprole on nutrient content

**Figure 6:**
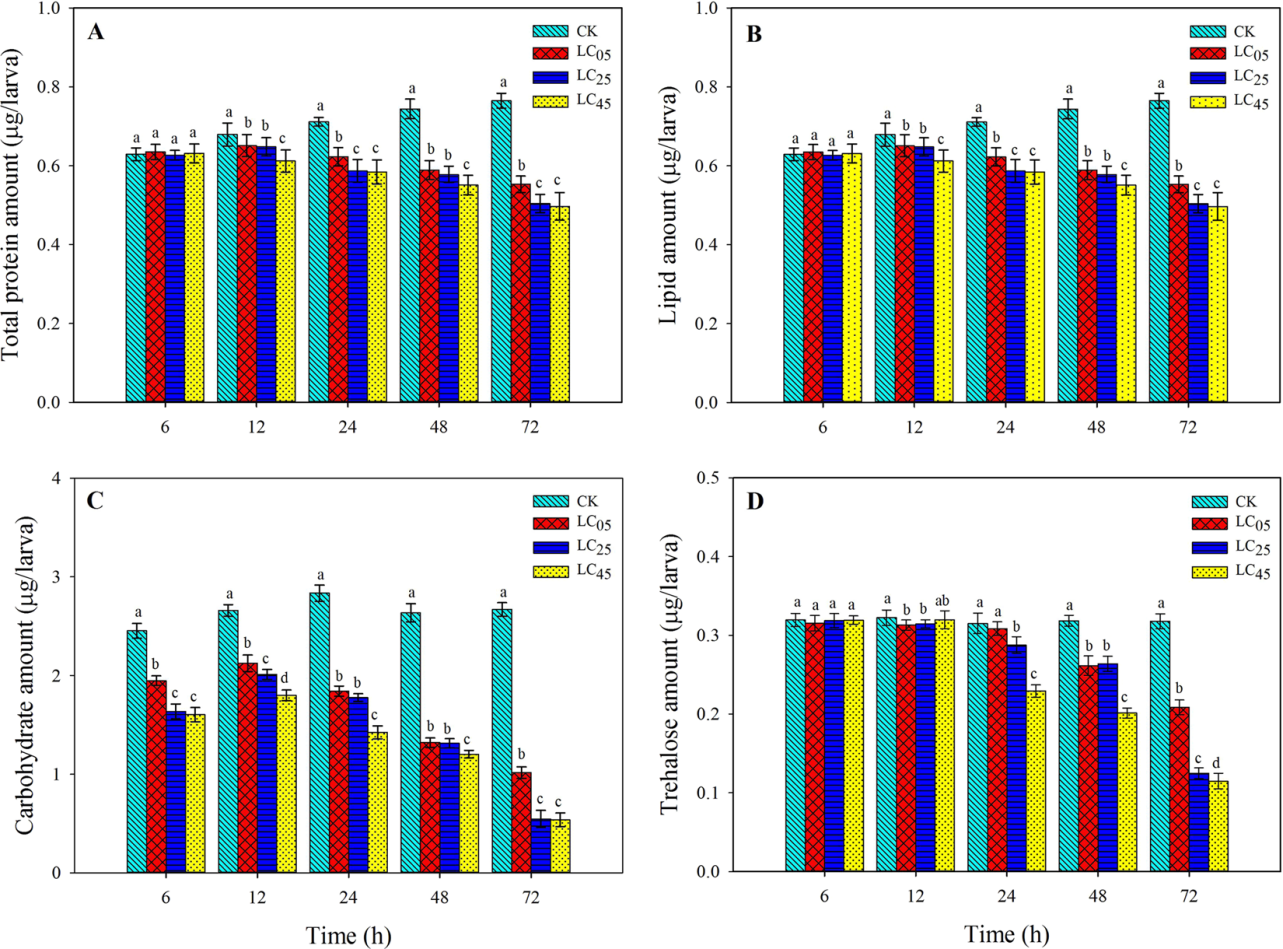
Nutrient content (**A**) total protein amount, (**B**) lipid amount, (**C**) carbohydrate amount, (**D**) trehalose amount) in *A. ipsilon* (mean ± SE) after after third-instar larvae exposure to low-lethal concentrations of chlorantraniliprole. Bar with the same lowercase letters indicate no significant differences (Student-Newman-Keuls test, *P* < 0.05).

The effects of chlorantraniliprole on the nutrient content in the third instar larvae of *A. ipsilon* are shown in Fig. 6. At low-lethal concentrations, chlorantraniliprole significantly decreased the protein and lipid content after treatment for 12, 24, 48, and 72 h compared with the control treatment (Fig. 6A,B). However, no significant effect was observed between the LC_05_, LC_25_ and LC_45_ treatments and the control after 1 h of treatment. The carbohydrate content was significantly decreased under the LC_05_, LC_25_ and LC_45_ treatments compared with the control (Fig. 6C). In addition, chlorantraniliprole did, to some extent, reduce the trehalose content when the third instar *A. ipsilon* larvae were treated with the three low-lethal concentrations of the insecticide (Fig. 6D).

### Effects of chlorantraniliprole on digestive enzyme activities

**Figure 7:**
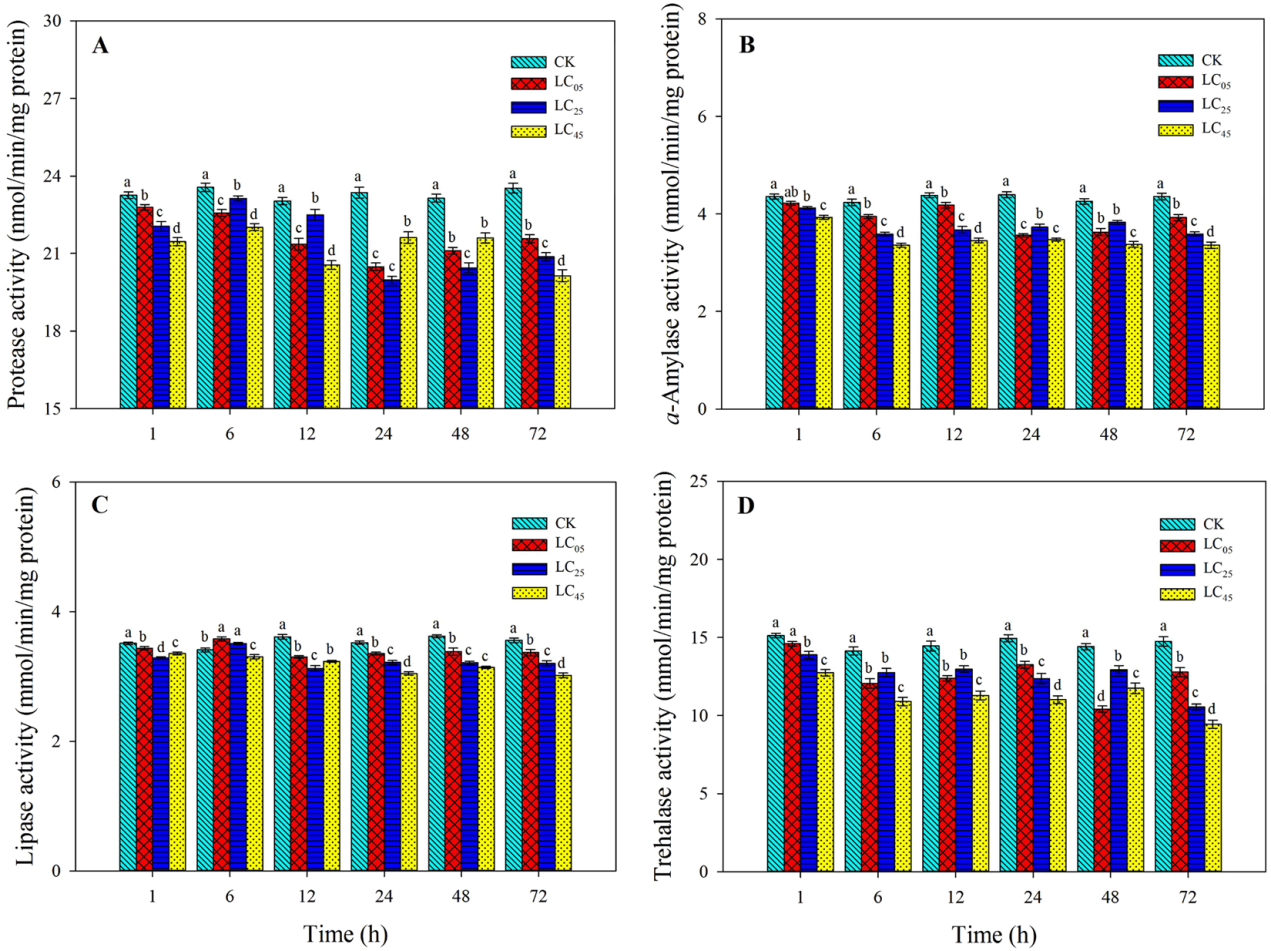
Digestive enzyme activities (**A**) protease, (**B**) α-amylase, (**C**) lipase, (**D**) trehalose) in *A. ipsilon* larvae (mean ± SE) after exposure to low-lethal doses of chlorantraniliprole. Bar with the same lowercase letters show no significant differences (Student-Newman-Keuls test, *P* < 0.05).

The effects of chlorantraniliprole on digestive enzyme activities in third instar *A. ipsilon* larvae are shown in Fig. 7. At low-lethal concentrations, chlorantraniliprole observably affected protease activity, α-amylase activity, or trehalase activity in *A. ipsilon* larvae compared to the effects of control treatment at 1, 6, 12, 24, 48, and 72 h (Fig. 7A,B and D). Lipase activity was significantly enhanced at 24 h after treatment with chlorantraniliprole at LC_05_ and LC_25_ compared to the activity with control treatment. However, only LC_45_ treatment significantly decreased lipase activity after treatment for 24 h compared to that of the control (Fig. 7C). Overall, the digestive enzyme activities decreased in a concentration-dependent manner.

### Effects of chlorantraniliprole on detoxifying enzyme activities

**Figure 8:**
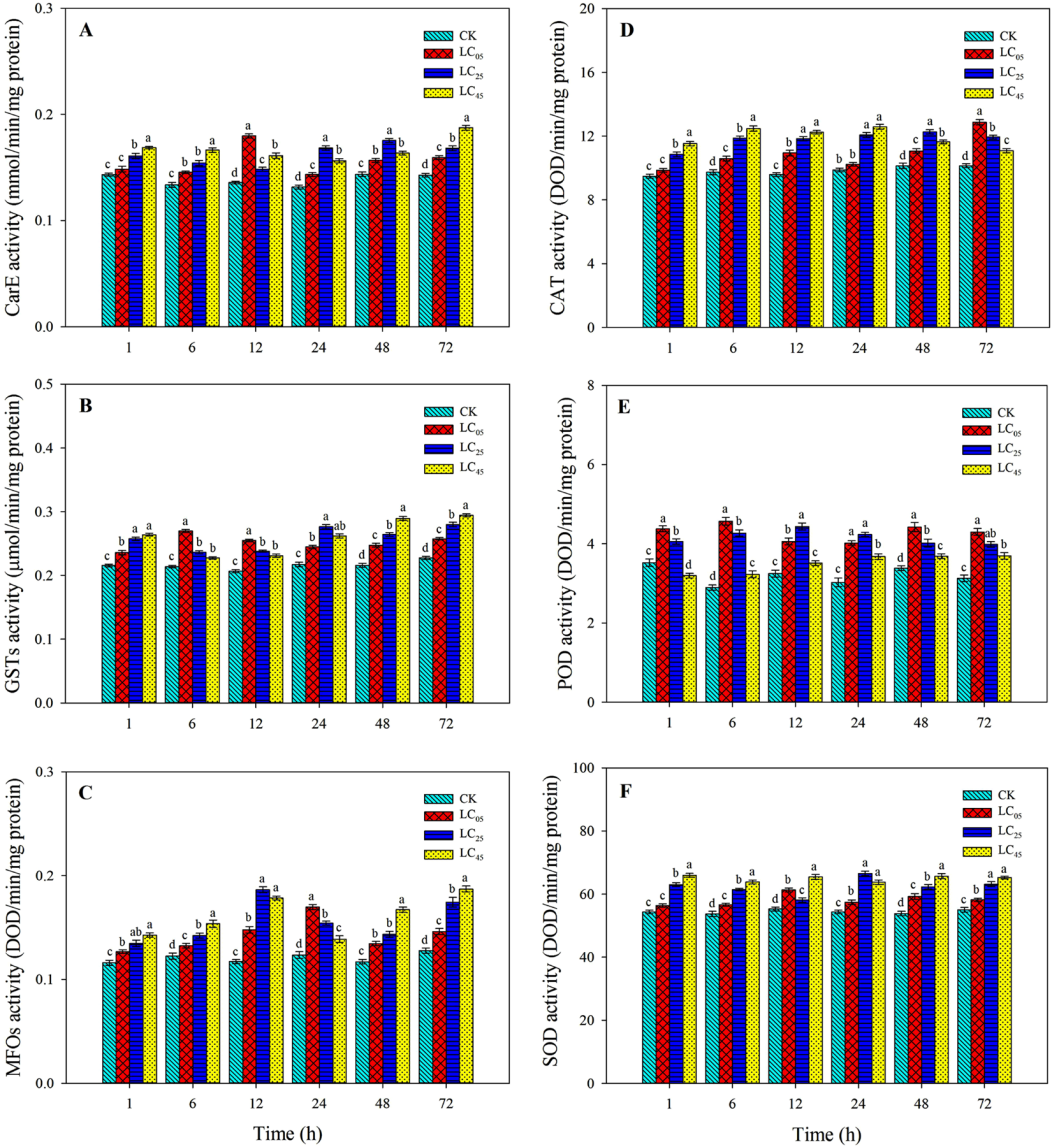
Detoxification enzyme activities (**A**) carboxyl esterase (CarE), (**B**) glutathione S-transferase (GSTs), (**C**) O-demethylation multi-function oxidase (MFOs)) and protective enzyme activities (**D**) catalase (CAT), (**E**) peroxidase (POD), (**F**) superoxide dismutase(SOD)) in third-instar larvae of *A. ipsilon* (mean ± SE) after exposure to low-lethal concentrations of chlorantraniliprole. Bar with the same lowercase letters indicate no significant differences (Student-Newman-Keuls test, *P* < 0.05).

The activities of detoxifying and protective enzymes were determined after third instar *A. ipsilon* larvae were treated with the three low-lethal concentrations of chlorantraniliprole (Fig. 8). After only 1 h, the carboxyl esterase activity (CarE) of the LC_05_ treatment exhibited no significant difference compared to the control; however, under the LC_25_ and LC_45_ treatments, the CarE activity increased significantly. Chlorantraniliprole significantly enhanced the activity of CarE after treatment for 6, 12, 24, 48, and 72 h (Fig. 8A). Glutathione S-transferase activity (GSTs) and multifunctional oxidase activity (MFOs) increased significantly under LC_05_, LC_25_ and LC_45_ chlorantraniliprole treatments after 1, 6, 12, 24, 48, and 72 h (Fig. 8B,C).

### Effects of chlorantraniliprole on protective enzyme activities

Catalase activity (CAT) and superoxide dismutase activity (SOD) significantly increased under all chlorantraniliprole treatments compared with those of the control group (Fig. 8D,F). After 1 h, the LC_45_ treatment significantly decreased the activity of peroxidase (POD), while POD activity increased dramatically after treatment for 6, 12, 24, 48, and 72 h compared with that of the control. At LC_05_ and LC_25_, chlorantraniliprole significantly enhanced the activity of POD compared to that of the LC45 treatment and control groups (Fig. 8E).

### Effects of chlorantraniliprole on hormone titers

**Figure 9:**
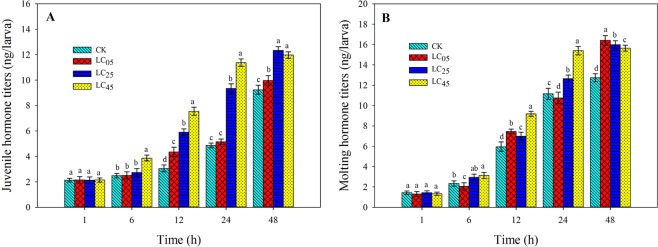
Hormone titers (**A**) juvenile hormone titers, (**B**) molting hormone titers) in *A. ipsilon* larvae after treatment with chlorantraniliprole. Bar with the same lowercase letters show no significant differences (Student-Newman-Keuls test, *P* < 0.05).

The effects of low-lethal concentrations of chlorantraniliprole on hormone titers in *A. ipsilon* larvae were determined (Fig. 9). The juvenile hormones (JH) and molting hormones (MH) titers clearly increased with growth and development of the third instar larvae of *A. ipsilon*. As the concentration increased, the JH and MH titers of the *A. ipsilon* larvae increased to some extent after exposure to the three treatment concentrations of chlorantraniliprole. However, there was no significant difference between the treatments and the control after chlorantraniliprole treatment for 1 h. The LC_05_ treatment significantly reduced the hormone titers after treatment for 6 h and 24 h compared to the control.

## Discussion

When selecting insecticides, in addition to acute toxicity, chronic toxicity against target pests should also be closely considered, as chronic toxicity could result in reduced survival, lifespan, growth and development, fecundity, and population growth after exposure to low doses of the toxic substance^26,27^. Insecticides with different modes and mechanisms of action can be applied to generate various effects against targeted insects at low-lethal concentrations^28,29^. Moreover, sublethal effects of the same insecticide on different types of target pests can also be significantly different^21,30^. In this study, we confirmed that chlorantraniliprole exhibits active toxicity against third instar larvae. Moreover, chlorantraniliprole at low-lethal doses considerably influenced the development, reproduction and demographic parameters of this cutworm. Many lepidopteran pests are also highly sensitive to this insecticide^7–9^, which has been registered for controlling many lepidopteran and non-lepidopteran pests in China^31^. Previous studies have shown that chlorantraniliprole can be used not only for foliar applications but also for seed treatment because it has excellent systemic properties and persistence^32,33^. Chlorantraniliprole could be applied for efficiency controlling the cutworm by seed treatment, which is an important strategy for insect pest management.

The larval stages of target insects can be significantly prolonged after exposure to low-lethal doses of insecticides^11^. We observed that chlorantraniliprole at low-lethal concentrations could also prolong *A. ipsilon* larval development (fifth and sixth stages). Previous studies have demonstrated that low doses of chlorantraniliprole could prolong the development time of many lepidopteran insect pests^10,34–36^. These results are consistent with those observed with the anthranilic diamide insecticide cyantraniliprole, which also prolonged the larval development of some lepidopteran pests^29,37^. We also found that the LC_05_ treatment of this insecticide could lengthen the durations of the pupal and adult stages. This effect was mainly due to the starvation caused by cessation of feeding, which led to the energy of the larvae being diverted to detoxification and metabolic processes rather than being used for individual growth and development^9,27^. The proportion of seventh-instar larvae attained increased when the *A. ipsilon* larvae were treated with this insecticide. The time needed to accumulate sufficient nutrients to maintain normal growth and development of *A. ipsilon* was longer when the third instar larvae were exposed to chlorantraniliprole than that needed with the control treatment.

Life table analysis is an effective tool for assessing the sublethal effects of insecticides on target insects at the population level, rather than at the individual level^38,39^. We found that low-lethal concentrations of chlorantraniliprole significantly impared the population parameters, including *R*_0_, *λ*, *r* and *T*, which are the most useful indicators of reproductive expectations for insect populations^40^. Chlorantraniliprole significantly extended the APOP and TPOP. The oviposition period was significantly prolonged under the LC_05_ treatment, while the LC_25_ and LC_45_ treatments significantly shortened the *A. ipsilon* female oviposition period. All the treatments resulted in a significant decrease in the total number of eggs laid by the female adults. Thus, these results indicated that female fecundity was significantly decreased after chlorantraniliprole treatment, largely because the mating times and oviposition period were greatly reduced. Similar studies have shown that chemicals can have adverse effects on female mating behavior and fecundity of target insects^41,42^. These results showed that low-lethal concentrations of chlorantraniliprole markedly inhibited *A. ipsilon* population growth.

Sublethal effects, such as hormesis the for F0 generation and selection for the F1 generation, of insecticides are considered to be common toxicological phenomena and could result in pest resurgence and secondary outbreaks^43,44^. The hormetic effects of insecticides have been described by Calabrese and Baldwin^45^ and stimulatory effects (low-dose stimulation and high-dose inhibition) have been observed in various insects^46,47^. Fecundity in adult females of target insect pests has been seen to significantly increase upon treatment with low-lethal concentrations of pesticides^46,47^. However, many studies have also shown that low doses of insecticides have adverse effects on pest reproduction^37^. The effects of hormesis are closely associated with insect species, pesticide mode of action and physiological states.

Demographic parameters, including the age-stage-specific survival rate (*s*_*xj*_), age-specific survival rate (*l*_*x*_), female age-specific fecundity (*f*_*x*9_), age-specific maternity (*l*_*x*_*m*_*x*_), female age-stage-specific reproductive value (*v*_*xj*_) and life expectancy (*e*_*xj*_) of *A. ipsilon* were reduced dramatically after exposure to chlorantraniliprole, which had a negative effect at the population levels. Nevertheless, the LC_25_ chlorantraniliprole treatment did, to some extent, increase the age-specific fecundity of the total population (*m*_*x*_), which was the same stimulatory effect as that observed for the *GRR*. This result proved that LC_25_ treatment may have had a certain stimulatory effect on the fecundity of the *A. ipsilon* population. However, no studies have shown that the *m*_*x*_ and *GRR* can be used to determine whether the target insects produce hormesis after exposure to pesticides. Therefore, chlorantraniliprole at low-lethal concentrations does not stimulate adult fecundity of *A. ipsilon*. In this study, we examined the sublethal effects of this chemical on only the parental generation of *A. ipsilon* under laboratory conditions. Therefore, field trials and long-term studies on offspring should be conducted in the future.

Feeding efficiency is an important aspect of feeding ecology, describing the ability of insects to convert ingested food to biomass and the effects of external and internal factors^48^. In our study, the AD was significantly reduced, largely due to feeding inhibition after the *A. ipsilon* larvae were treated with chlorantraniliprole. Nathan *et al*. showed that retarded movement of food in the digestive tract and prolonged larval development time resulted in increased AD values^49^. This finding is similar to our results, in which the development time of *A. ipsilon* larvae was lengthened after exposure to low-lethal doses of chlorantraniliprole. Stoyenoff *et al*. found that the high AD was mainly due to the passage of food through the digestive tract at a decreased rate when the target insect had consumed a low amount of food^50^. With larval growth, treatment with this chemical also led to significant reduction in the MRGR, which is an index that is closely associated with insect weight, with increasing concentration of insecticide^29^. Chlorantraniliprole reduced the pupal weight; however, no significant difference was observed in pupal weight after exposure to low-lethal doses of the insecticide. Similarly, the MRGRs of *Cnaphalocrocis medinalis* were dramatically reduced after fourth-instar larvae were treated as described by Nathan^49^. Liu *et al*. also found that fraxinellone greatly decreased the MRGRs of *Ostrinia furnacalis*^51^. In contrast, the ECI and ECD significantly increased with increasing concentrations of this chemical. Previous work has demonstrated that insecticides and plant extracts have negative effects on the feeding efficiency of targeted insects^48,52^. These findings suggest that artificial diets treated with insecticides may act as inhibitors by denying the sufficient nutrients for normal growth and metabolic processes. This phenomenon may be directly associated with the low survival rate, developmental delay and reproduction of the *A. ipsilon* population after exposure to chlorantraniliprole.

Proteins, carbohydrates and lipids are important chemical constituents of insect bodies that provide the necessary energy to maintain bodily functions and participate in the metabolic processes of many physiological and biochemical reactions^29,38^. In this study, chlorantraniliprole at low doses significantly reduced the protein content. When protein levels are low, metabolic processes may be disrupted by the degradation of proteins to amino acids and by the TCA cycle compensating for the decrease in energy caused by pesticide stress^53^. Protease activity was significantly reduced after exposure to the chemical. This result was consistent with another report, which showed that clitocypin inhibits protease activity in *Leptinotarsa decemlineata* (Say)^54^. Protease activity in *Bradysia odoriphaga* was decreased upon treatment with benzothiazole^21^ and chlorfenapyr^38^. The carbohydrate content was markedly decreased when *A. ipsilon* was treated with chlorantraniliprole, which may be due to reduction of food consumption. Simultaneously, α-amylase activity was also significantly reduced after exposure to the insecticide, resulting in reduced conversion of glycogen to glucose and hydrolysis of starch to maltose after chlorantraniliprole treatment^21^. These results were consistent with the reports of Etebari *et al*.^55^ and Yazdani *et al*.^48^ and may also explain the decrease in carbohydrate content. In addition, chlorantraniliprole significantly affected the lipid content and lipase activity, which may be associated with the disruption of lipid metabolism and hormone secretion after treatment with the chemical^56^. Zibaee *et al*. found that the activity of lipase in *Eurygaster integriceps* was reduced after exposure to *Artemisia annua* extract^57^. Many studies have also demonstrated that chemical insecticide compounds affect lipid content and lipase activity^29,38^. The levels of these macronutrients were reduced in the body when the feeding efficiency of the target insects was affected by insecticides, and this effect varied among feeding conditions and different growth stages^38^. Upon insecticide treatment, the normal consumption and digestion processes are affected, mainly due to imbalance of the secretion and function of digestive enzymes in target insects^57^.

Trehalose is the main blood sugar in the hemolymph of insects and is an important energy source in many physiological activities, such as metamorphosis, flight and reproduction, regulating trehalose metabolism and controlling the utilization of glucose^29,58^. Trehalose can effectively prevent protein denaturation and loss of function under adverse conditions^38^. In this study, trehalose content and trehalase activity decreased significantly after exposure to chlorantraniliprole. Previous studies have demonstrated an increase in the proportion of trehalose among total carbohydrates to enhance the ability to adapt to insecticidal stress^58^. This effect is similar to those of benzothiazole and chlorfenapyr on *B. odoriphaga*^29,38^ and that of hexaflumuron on *Apolygus lucorum*^59^. In addition, the trehalose content can also affect the food selection and feeding behavior of insects^60^. Therefore, trehalose plays a very important role in the regulation of the growth and development of target insects.

Insecticides not only affect the growth and development, fecundity and population parameters of target insects but also disrupt the activities of protective enzymes and detoxifying enzymes^38,61^. In the present study, chlorantraniliprole at low-lethal concentrations significantly increased the activities of CAT and SOD in *A. ipsilon* larvae at all the studied time points compared with the control groups, which was consistent with the results of Zhang *et al*.^62^ POD activity was initially inhibited and subsequently activated after exposure to the LC_05_ treatment of chlorantraniliprole. The LC_25_ and LC_45_ treatments significantly induced SOD activity. CAT, POD and SOD are three major protective enzymes that maintain a state of dynamic equilibrium, protecting target insects from free-radical attacks^62,63^. Therefore, activation or inhibition of SOD, CAT and POD activities by insecticide treatment could disrupt the physiological metabolic homeostasis and ultimately lead to growth inhibition and insect death.

Detoxifying enzymes are the main enzymes involved in the metabolism of toxic substances in insects^38,61^. In this study, the activities of CarE and GSTs increased significantly in the chlorantraniliprole treatments compared to the control groups. Previous studies have demonstrated that CarE and GSTs are the primary enzymes involved in the metabolism of xenobiotics (e.g., insecticides) in target insects^29,38,64^. In addition, chlorantraniliprole at low-lethal doses greatly increased the MFO activity compared to the control. A significant difference was observed between the treatments and the control, and this difference increased as the concentration of the chemical increased. These results were consistent with those of Zhang *et al*.^62^. and Devorshak *et al*.^65^. Furthermore, the detoxification process requires a large amount of energy, which may lead to reduced nutrient content in insects, as observed in this study. This finding has also been confirmed in reports by Zhao *et al*.^29^, Devorshak *et al*.^65^ and Yazdani *et al*.^48^. In addition, Zhao *et al*. found that these enzymes allow the treated insects to survive, and the activities of these enzymes may lead to an increase in total protein content after insecticide treatment^29^. However, this result was different from the results of this study, which may be partly due to the unique mechanism of action of chlorantraniliprole. These results indicated that these enzymes could play a role in chlorantraniliprole metabolism and detoxification.

MH and JH regulate many physiological processes (e.g., molting, ecdysis, reproduction and behavior) during the growth and development of target insects^52^. In the present study, no significant difference was observed between the chlorantraniliprole treatments and the control after treatment for 1 h; however, the MH and JH titers significantly increased after treatment with the chemical with increasing treatment time. Previous research has suggested that JH and MH titers vary among insect stages and host plant species^66^. The development time could be delayed by alteration of the hormone titers in insects after insecticide treatment^52^. Chlorantraniliprole at low doses prolonged not only larval development but also pupal development. However, previous findings have shown that MFOs metabolize and decrease the levels of JHs and JH analogs, which may result in shortening of the development time of target insects by insecticide treatment^62,67^. This study shows that the mechanism of action of chlorantraniliprole on *A. ipsilo*n is complex, and further genomic and proteomics studies are required.

## Materials and Methods

### Insect rearing and insecticide

*A. ipsilon* adults were originally collected from a cornfield in Ningyang City (35.76N, 116.80E) in the Shandong Province of China during June 2012. After collection, adult moths were transferred to a spawning container (30 cm L × 15 cm W × 15 cm H) and provided with a 20% honey solution as food. The eggs were collected daily and moved to an incubation container (15 cm L × 15 cm W × 10 cm H) to hatch.

Newly hatched larvae were transferred into 24-well cell culture plates (Jet, Biofil, TCP010024) and reared with fresh leaves of pesticide-free cabbage (*Brassica oleracea* L.) before third instar larvae developed. Then, third instar and/or higher instar larvae were reared individually in 12-well cell culture plates (Jet, Biofil, TCP010012) to prevent cannibalism and were given an artificial diet^68^. Larvae were transferred into finger-shaped glass tubes (2.2-cm diam., 8 cm H) at the last larval instar so that larvae could molt normally into the pupal stage. All pupae were collected and then shifted into a spawning container after emergence. To maintain genetic diversity of the colony, feral *A. ipsilon* adults were collected in local cornfields annually. Rearing was maintained at 28 ± 1 °C, with 75 ± 5% relative humidity (RH) and a photoperiod of 16:8 h (L: D) in a climate-controlled room.

The chlorantraniliprole (95.3% technical grade) in this study was supplied by Shanghai DuPont Agrochemical Co., Ltd., China. Molting hormone (95%, MH) and juvenile hormone (93%, JH) were purchased from Sigma Chemical Co., USA.

### Lethal effects of chlorantraniliprole on third instar larvae of *A. ipsilon*

The bioassay method to assess the effects of chlorantraniliprole on newly molted third instar larvae (<12 h) of *A. ipsilon* was determined by mixing an artificial diet with insecticide. The active ingredient was dissolved with analytical grade acetone at a concentration of 100 mg/L. In a preparatory test to find the effective dose range, a series of desired chlorantraniliprole concentrations were prepared by diluting with distilled water. Chlorantraniliprole was added to the diet and mixed thoroughly to obtain concentrations of 4.0, 2.0, 1.0, 0.5, 0.25, and 0.125 μg.g^−1^; the temperature of the artificial diet was dropped below 50 °C during preparation processes. The control treatment was mixed with distilled water. The diet was cut into 1-cm^3^ cubes.

The third instar larvae (<12 h) of the same batch were transferred into 12-well cell culture plates and starved 3–4 h before toxicity tests. Larvae were fed with the artificial diet treated with different concentrations of chlorantraniliprole. For each treatment, 120 third instar larvae were used, and the bioassay employed a completely randomized design with seven treatments and five replicates. Larvae that showed extreme shrinkage compared with that of the control animals or larvae that were unable to move normally when stimulated with a writing brush after 72 h of treatment were considered dead.

### Sublethal effects of chlorantraniliprole on the growth and population parameters of *A. ipsilon*

To assess the sublethal effects of chlorantraniliprole on *A. ipsilon*, artificial diets containing chlorantraniliprole at LC_05_, LC_25_ and LC_45_ and distilled water for the control were prepared. Newly molted third instar larvae were selected and each individual larva was considered one replicate. The tested larvae were fed an insecticide-treated diet (at LC_05_, LC_25_, LC_45_) or an untreated diet (as a control) prepared in advance as described above.

The surviving *A. ipsilon* larvae from all the treatments were selected and individually transferred into clean 12-well cell culture plates after 72 h of treatment. The larvae were reared with an artificial diet without chlorantraniliprole treatment provided *ad libitum* and monitored daily until they reached the pupal stage. Pupae that did shrink, soft and rigidity of body were considered deformity. All pupae were individually shifted into finger-shaped glass tubes until adult insects emerged. The pupae (two days old) were weighed using an electronic analytical balance (Sartorius, BT125D, Germany). Adults that did deficiency and asymmetry of wings were considered deformity. Newly emerged *A. ipsilon* adults were collected from the glass tubes, sexed and paired (one female with one male) in a spawning container (10 cm diam. × 10 cm height), and 20% honey solution was provided as food to the adult moths. The oviposition container containing adults was checked daily for oviposition after eggs were first observed. Eggs were collected daily on Kraft paper, which served as an oviposition substrate and was replaced with new Kraft paper daily. All eggs were counted on the next day with a magnifying glass. Eggs that did dehydrated and absence of pedicel were considered deformity. During the experiment, all artificial diets and the 20% honey solution were kept fresh to ensure normal growth of larvae and adults.

### Effects of chlorantraniliprole on nutritional indices of *A. ipsilon* larvae

To evaluate the effects of chlorantraniliprole on food consumption and utilization by *A. ipsilon* larvae, artificial diets containing chlorantraniliprole at the LC_05_, LC_25_, and LC_45_ concentrations were prepared in advance. An artificial diet treated with distilled water was used as the control. The weights of all tested insects and food provided in this experiment were determined using an electronic analytical balance. Then, the third instar larvae were reared as described above. After treatment for 72 h, the surviving larvae and any remaining artificial diet and fecal matter produced were separated and weighed. To compensate for the decrease in weight of the food provided to the larvae because of evaporation, we simultaneously conducted a control experiment by maintaining weighed diets in 12-well cell culture plates and reweighed these diets after 72 h. Each of 20 larvae were tested per replicate, and six replicates we examined for each treatment. This experiment was conducted for 72 h. The food utilization rates were estimated according to the following formulas^69^:

$$({\rm{I}})\,{\rm{Mean}}\,{\rm{relative}}\,{\rm{growth}}\,{\rm{rate}}\,({\rm{MRGR}})=(\mathrm{log}\,{W}_{2}-\,\mathrm{log}\,{W}_{1})/T;$$

$$({\rm{II}}){\rm{Approximate}}\,{\rm{digestibility}}\,({\rm{AD}})=[(Q-F)/Q]\times 100;$$

$$({\rm{III}}){\rm{Efficiency}}\,{\rm{of}}\,{\rm{conversion}}\,{\rm{of}}\,{\rm{ingested}}\,{\rm{food}}\,({\rm{ECI}})=[(\mathrm{log}\,{W}_{2}-\,\mathrm{log}\,{W}_{1})/Q]\times 100;$$

$$({\rm{IV}}){\rm{Efficiency}}\,{\rm{of}}\,{\rm{conversion}}\,{\rm{of}}\,{\rm{digested}}\,{\rm{food}}({\rm{ECD}})=[({W}_{2}-{W}_{1})/(Q-F)]\times 100,$$

where *W*_1_ = the weight of larvae before treatment (g); *W*_2_ = the weight of larvae after treatment (g); *T* = the duration of the experimental period (days); *Q* = the fresh weight gain of larvae during the experimental period (g); and *F* = the mass of fecal matter produced by larvae during the experimental period (g).

### Effects of chlorantraniliprole on the nutrient content of *A. ipsilon*

For preparation of samples for physiological and biochemical assays, newly molted third instar larvae (<12 h) were fed artificial diets containing chlorantraniliprole at the LC_05_, LC_25_ and LC_45_ concentrations (as described above). The surviving larvae were selected and weighed after treatment. Larvae were placed in individual 2.0-mL centrifuge tubes and treated with liquid nitrogen, and then the samples were stored at −80 °C for preservation. One larva was tested per replicate, and 20 replicates were examined for each treatment. All procedures were carried out in an ice bath when the enzyme sources were prepared.

### Carbohydrate content

Individual larvae were homogenized in 500 μL of 10% trichloroacetic acid (TCA) on ice and centrifuged at 20,000 g force for 10 min at 4 °C. For each sample, 30 μL of the supernatant was mixed with 70 μL of 10% TCA. Then, 600 μL of 0.2% anthrone (200 mg of anthrone dissolved in 100 mL of 98% H_2_SO_4_) was added to each sample tube, which was then heated in a thermostat-controlled water bath at 90 °C for 10 min. The absorbance was measured at 630 nm using the BioTek Synergy^TM^ 2 Multi-Mode Reader (BioTek Instruments, Inc. Winooski, Vermont, USA). The carbohydrate content of all the samples was quantified based on the standard curve for glucose.

### Lipid content

Individual larvae were homogenized in 200 μL of 2% Na_2_SO_4_. Lipids were extracted with 750 μL of methanol:chloroform (1:2) for 4 h. Then, the mixture was centrifuged at 20,000 g force for 10 min at 4 °C. Five hundred microliters of the supernatant of each sample was transferred into a new tube and dried at 40 °C for 12 h. All samples were treated with 500 μL 98% H_2_SO_4_ individually and incubated in a water bath at 90 °C for 10 min. Finally, 30 μL of each sample was mixed with 270 μL of vanillin reagent (6 mg of vanillin dissolved in 1 mL of distilled water and 4 mL of 85% H_3_PO_4_). The absorbance was measured at 530 nm after 30 min, and the standard curve was prepared using cholesterol.

### Trehalose content

Individual larvae were homogenized with 100 μL of 20 mM phosphate buffer (pH 5.8) on ice, followed by centrifugation at 20,000 g force at 4 °C for 10 min. Then, 30 μL of the supernatant was mixed with 30 μL of 1% H_2_SO_4_ and incubated in a thermostat-controlled water bath at 90 °C for 10 min. After cooling on ice for 3 min, 30 μL of 30% KOH was added to each sample, and the mixture was incubated again at 90 °C for 10 min. Next, 600 μL of 0.2% anthrone was added after cooling for 3 min on ice, and the mixtures were incubated again at 90 °C for 10 min. After cooling on ice, the absorbance was measured at 630 nm, and trehalose was used to establish the standard curve, which was used for determining the trehalose content.

### Total protein content

The total protein content was determined according to the method developed by Bradford (1976)^70^. Individual larvae were homogenized in 600 μL of 50 mM Tris-HCl (pH 7.1; containing 0.5% Triton X-100 and 20% sucrose) on ice, and then the samples were centrifuged at 20,000 g force at 4 °C for 10 min. Then, 30 μL of the supernatant was subsequently mixed with 150 μL of 0.01% Coomassie Brilliant Blue G-250 as a dye. Then, the absorbance was measured at 595 nm using a BioTek Synergy^TM^ 2 multimode reader. The total protein content was calculated using a standard curve based on bovine serum albumin.

### Effects of chlorantraniliprole on *A. ipsilon* digestive enzyme activities protease activity

Individual larvae were homogenized with 100 μL of 20 mM phosphate buffer (pH 7.0) on ice, followed by centrifugation at 20,000 g force at 4 °C for 10 min. Casein was used as the substrate. Then, 30 μL of the supernatant was mixed with 100 μL of 1% casein and incubated in a water bath at 37 °C for 15 min. Next, this reaction was stopped via the addition of 100 μL of 0.4 M TCA, followed by centrifugation at 20,000 g force at 4 °C for 10 min. A 100 μL aliquot of the supernatant was subsequently transferred to a new tube and mixed with 500 μL of 0.4 M Na_2_CO_3_ and 100 μL of Folin-Ciocalteu reagent, after which, the sample was incubated at 37 °C for 30 min, and the absorbance was measured at 660 nm. Tyrosine was used to generate the standard curve.

### Lipase activity

The crude enzyme was extracted as described in protease activity. Then, 10 μL of the enzyme solution was mixed with 18 μL of 50 mM 4-nitrophenyl butyrate and 172 μL of phosphate buffer (pH 7.0) and incubated in a water bath at 37 °C for 15 min. Next, the mixture was transferred to −20 °C for 5 min to stop the reaction. The absorbance was measured at 405 nm, and p-nitrophenol was used to establish the standard curve.

### *α*-Amylase activity

First, the crude enzyme was extracted with 20 mM sodium phosphate buffer (pH 6.9) on ice, followed by centrifugation at 20,000 g force at 4 °C for 10 min. Next, 10 μL of the supernatant was mixed with 40 μL of 1% soluble starch and 50 μL of phosphate buffer, and the reaction was maintained in a water bath at 35 °C for 30 min. Then, the reaction was stopped by adding 100 μL of dinitrosalicylic acid (DNS) and heating in boiling water for 10 min. After cooling on ice, the absorbance was measured at 540 nm, and the standard curve was established using maltose.

### Trehalase activity

First, the crude enzyme was extracted with 20 mM phosphate buffer (pH 5.8). In each tube, 30 μL of the supernatant was mixed with 60 μL of 40 mM trehalose, followed by incubation at 37 °C for 30 min. Then, the reaction was arrested by heating in boiling water for 3 min and cooling on ice. Next, 90 μL of an indicator dye (5 g of 3,5-dinitrosalicylic acid, 5 g of NaOH, 1 g of phenol and 0.25 g of anhydrous sodium sulfite dissolved in 500 mL of distilled water) was added to each tube, and the mixture was incubated in a water bath at 90 °C for 5 min. After cooling on ice, 30 μL of potassium sodium tartrate was added, and the absorbance was measured at 550 nm. The standard curve was established using glucose.

### Effects of chlorantraniliprole on *A. ipsilon* detoxifying enzyme activities carboxyl esterase activity (CarE)

Individual larvae were homogenized in 100 μL of 40 mM phosphate buffer (pH 7.0) on ice, followed by centrifugation at 20,000 g force at 4 °C for 10 min. Then, 25 μL of the supernatant was mixed with 100 μL of 0.03 M α-naphthyl acetate, which was used as a substrate for the enzymatic reaction, and 1 mL of 1 × 10^−4^ M eserine, which was diluted with 40 mM phosphate buffer (pH 7.0) to 100 mL. The mixtures were incubated in a 37 °C water bath for 10 min. Next, the reactions were stopped by the addition of 75 μL of stopping solution (1% Fast Blue B salt solution and 5% sodium dodecyl sulfate solution mixed at a ratio of 2:5). The absorbance was measured at 600 nm after 30 min, and *α*-naphthol was used to generate the standard curve.

### Glutathione *S*-transferase activity (GSTs)

Individual larvae were homogenized in 100 μL of 66 mM buffer (pH 7.0; 58 mg of EDTA in 66 mL of 0.1 M PBS (pH 7.0) and 34 mL of double-distilled water) on ice, followed by centrifugation at 20,000 g force at 4 °C for 10 min. Then, 10 μL of the supernatant was mixed with 5 μL of 30 mM 2,4-dinitrochlorobenzene (DNCB) and 15 μL of 50 mM glutathione. The control group was not treated with the enzyme extract. The absorbance was measured at 340 nm for 15 min. Enzyme activity was expressed in μmol/min/mg protein.

### Multifunctional oxidase activity (MFOs)

Individual larvae were homogenized in 200 μL of 0.1 M phosphate buffer (pH 7.0; containing 1 mM dithiothreitol, 1 mM ethylene diamine tetraacetic acid, 0.4 mM phenylmethanesulfonyl fluoride, and 1 mM propylthiouracil on ice, followed by centrifugation at 20,000 g force at 4 °C for 10 min. Then, 90 μL of the supernatant was mixed with 100 μL of 2 mM p-nitroanisole (substrate) for 2 min. Next, 10 μL of 9.6 mM triphosphopyridine nucleotide was added, and the resulting mixture was immediately placed in a microplate reader. The absorbance was measured at 405 nm once per minute for 30 min. Enzyme activity was expressed in ΔOD/min/mg protein.

### Effects of chlorantraniliprole on *A. ipsilon* protective enzyme activities peroxidase activity (POD)

Individual larvae were homogenized in 200 μL of precooled 1% polyvinylpyrrolidone solution on ice, followed by centrifugation at 20,000 g force at 4 °C for 10 min, and the supernatant was used as the enzyme source. Next, a 4-mL reaction system was prepared, containing 200 μL of enzyme source, 1.8 mL of acetate buffer (pH 5.0), 1 mL of 0.1% guaiacol and 1 mL of 0.08% H_2_O_2_. The absorbance was measured at 470 nm at 30 °C for 30 min using a BioTek Synergy^TM^ 2 multimode reader. Enzyme activity was expressed in ΔOD/min/mg protein.

### Catalase activity (CAT)

The protocol for enzyme source extraction was consistent with those of POD activity. The reaction system contained 2 mL of pH 7.0 phosphate buffer, 1 mL of 0.08% H_2_O_2_ (the blank instead used 1 mL of distilled water) and 100 μL of enzyme source. Absorbance was read at 240 nm at 30 °C for 30 s using the BioTek Synergy^TM^ 2 Multi-Mode Reader. Enzyme activity was expressed in ΔOD/min/mg protein.

### Superoxide dismutase activity (SOD)

Extraction of enzyme source was performed as described in POD activity. A 3-mL reaction system was prepared, containing 20 μL of enzyme source, 80 μM riboflavin, 77 μM nitro-blue tetrazolium (NBT), 13 mM methionine and 0.1 mM EDTA. The reaction was performed by illuminating at 4000 l for 5 min and stopped by placing in the dark. Then, the absorbance was measured at 560 nm at 25 °C using a BioTek Synergy 2 multimode reader. Suppression of NBT to 50% was considered to be one unit of enzyme activity (U). Enzyme activity was expressed in ΔOD/min/mg protein.

### Effects of chlorantraniliprole on hormone titers of *A. ipsilon* Molting hormone (MH)

First, MH was accurately weighed and dissolved in methanol solution (1:1) as a stock solution. Then, standard solutions with concentrations of 55.5 ng·L^−1^, 27.75 ng·L^−1^, 13.875 ng·L^−1^, 6.9375 ng·L^−1^, 3.4688 ng·L^−1^, and 1.7344 ng·L^−1^ were prepared to obtain a standard curve. The standard solutions were used as the abscissa, and the peak areas were used as the ordinates. Individual larvae were weighed and homogenized with 2 mL of methanol (75%) in a glass homogenizer, followed by centrifugation at 20,000 g force for 10 min. Samples were extracted three times with methanol (75%) and dried completely using a pressure blowing concentrator. Next, the MH samples were dissolved in 2 mL of double-distilled water and subsequently transferred into 5 mL of chloroform. The water and chloroform (1:1) were thoroughly mixed and allowed to separate for 5 min under static conditions after the chloroform was fully mixed with the water. The upper layer was concentrated by placing the solution in a water bath at 60 °C. Then, 150 μL of standard solution was added to 150 μL of mobile phase, and the obtained solution was subsequently filtered using an organic phase membrane before being subjected to chromatographic analysis. Chromatographic conditions were described as Supplementary Information.

### Juvenile hormone (JH)

First, JH was accurately weighed and dissolved in absolute ethanol (≥99.70%) as a stock solution. Then, a standard curve was prepared by serially diluting the stock solution (62.5 ng·L^−1^, 31.25 ng·L^−1^, 15.625 ng·L^−1^, 7.8125 ng·L^−1^, 3.9061 ng·L^−1^, and 1.9531 ng·L^−1^) to prepare standard solutions. Individual larvae were weighed and homogenized with 2 mL of methanol and ether (1:1) in a glass homogenizer, followed by centrifugation at 20,000 g force for 10 min. Samples were extracted three times with n-hexane. The upper phase was collected and dried completely at 40 °C under nitrogen gas. Next, 150 μL of standard solution was added to 150 μL of mobile phase, and the obtained solution was subsequently filtered using an organic phase membrane before being subjected to chromatographic analysis. Chromatographic conditions were described as Supplementary Information.

### Data analysis

The data obtained from the toxicity test were corrected using Abbott’s formula^71^ before analysis, and data were subjected to probit analysis using SPSS (version 19.0, SPSS Inc., Chicago, IL, USA). Statistically significant mean values were determined using ANOVA, and the significant differences among the treatments were determined using the Student-Newman-Keuls test (*P* < 0.05).

Raw population parameter data for all *A. ipsilon* individuals were analyzed based on the age-stage, two-sex life table theory^50,72^. The age-stage-specific survival rate (*s*_*xj*_) represents the probability that each egg can survive to age *x* and stage *j*; the female age-specific fecundity (*f*_*x*_9) means the number of eggs produced by a female adult at age *x*; the curve for age-stage-specific reproductive values (*v*_*xj*_) represents the contribution of each individual to future offspring from age *x* to stage *j*. The age-specific survival rate (*l*_*x*_) is a simplified form of *s*_*xj*_, and age-specific fecundity (*m*_*x*_) is calculated as follows:

$${l}_{x}=\sum _{{j}=1}^{{\beta }}{sxj}$$

and

$${m}_{x}=\frac{{\sum }_{j=1}^{\beta }sxjfxj}{{\sum }_{j=1}^{\beta }sxj}$$

The net reproductive rate (*R*_0_) was calculated as follows:

$${R}_{0}=\sum _{{j}=1}^{\infty }{{l}}_{{x}}{m}_{x}$$

The intrinsic rate of increase (*r*) was calculated as follows:

$$\sum _{x=0}^{\infty }{e}^{-r(x+1)}{l}_{x}{m}_{x}=1$$

The mean generation time (*T*) was calculated as follows:

$$T={\rm{In}}\,{R}_{0}/r$$

The finite rate of increase (*λ*) was calculated as follows:

$$\lambda ={e}^{r}$$

Developmental time, fecundity and longevity and their mean values, standard error (SE) and differences among treatments were estimated by the bootstrap test method (B = 200,000)^73,74^ included in the computer program TWOSEX-MSChart^10,31^. Population parameters were also analyzed, and their mean values, SE values and significant differences were determined using the bootstrap method included in the computer program TWOSEX-MSChart^73,74^. All curves for survival rates, fecundity, reproductive value, and life expectancy were constructed using SigmaPlot 12.5 (Systat, Erkrath, Germany).

## Supplementary information

SUPPLEMENTARY INFORMATION
