# Supplementary material for: Chlorantraniliprole against the black cutworm Agrotis ipsilon (Lepidoptera: Noctuidae): From biochemical/physiological to demographic responses
Source: Sci Rep. 2019 Jul 17;9:10328. doi: 10.1038/s41598-019-46915-0 (PMC6637144; doi:10.1038/s41598-019-46915-0)
Supplement: Supplementary file 1 — SUPPLEMENTARY INFORMATION [file 41598_2019_46915_MOESM1_ESM.doc]

**Chlorantraniliprole against** **the black cutworm *Agrotis ipsilon* (Lepidoptera: Noctuidae): From biochemical/physiological to demographic responses**

Falin He^1,2^, Shiang Sun^1^, Haili Tan^1^, Xiao Sun^1^, Chao Qin^2^, Shoumin Ji^2^, Xiangdong Li^3^, Jiwang Zhang^4^ & Xingyin Jiang^1, 2^

^1^Key Laboratory of Pesticide Toxicology and Application Technique, College of Plant Protection, Shandong Agricultural University, Tai’an Shandong 271018, China. ^2^Research Center of Pesticide Environmental Toxicology, Shandong Agricultural University, Tai’an, Shandong 271018, China.

^3^Shandong Provincial Key Laboratory of Agricultural Microbiology, College of Plant Protection, Shandong Agricultural University, Tai’an Shandong 271018, China.

^4^State Key Laboratory of Crop Biology, College of Agronomy, Shandong Agricultural University, Tai’an Shandong 271018, China.

Falin He and Shiang Sun contributed equally to this work.

Correspondence and requests for materials should be addressed to X.J. (email: xyjiang@sdau.edu.cn)

**Chromatographic conditions**

**Molting hormone (MH):** mobile phase, methanol and water (50:50); flow rate, 0.5 mL·min^−1^; detection wavelength, 254 nm; injection volume, 10 μL; and column temperature, 24℃.

**Juvenile hormone (JH):** mobile phase, methanol and water (70:30); flow rate, 1 mL·min^−1^; detection wavelength, 218 nm; injection volume, 10 μL; and column temperature, 24℃. The peak area was quantifie.
